# MhcVizPipe: A Quality Control Software for Rapid Assessment of Small- to Large-Scale Immunopeptidome Datasets

**DOI:** 10.1016/j.mcpro.2021.100178

**Published:** 2021-11-17

**Authors:** Kevin A. Kovalchik, Qing Ma, Laura Wessling, Frederic Saab, Jérôme D. Duquette, Peter Kubiniok, David J. Hamelin, Pouya Faridi, Chen Li, Anthony W. Purcell, Anne Jang, Eustache Paramithiotis, Marco Tognetti, Lukas Reiter, Roland Bruderer, Joël Lanoix, Éric Bonneil, Mathieu Courcelles, Pierre Thibault, Etienne Caron, Isabelle Sirois

**Affiliations:** 1 CHU Sainte-Justine Research Center, Montreal, Quebec, Canada; 2 School of Electrical Engineering and Computer Science, Faculty of Engineering, University of Ottawa, Ontario, Canada; 3 Infection and Immunity Program and Department of Biochemistry and Molecular Biology, Biomedicine Discovery Institute, Monash University, Clayton, Victoria, Australia; 4 CellCarta, Montreal, Quebec, Canada; 5 Biognosys, Schlieren, Switzerland; 6 Institute of Research in Immunology and Cancer, Montreal, Quebec, Canada; 7 Department of Chemistry, Université de Montréal, Montreal, Quebec, Canada; 8 Department of Pathology and Cellular Biology, Faculty of Medicine, Université de Montréal, Quebec, Canada

**Keywords:** MHC, peptide, immunopeptidomics, software, MS, BA, binding affinity, BF, binding fraction, CLI, command line interface, EL, eluted ligand, GUI, graphical user interface, HLA, human leukocyte antigen, KLD, Kullback-Leibler Distance, LF, length fraction, MHC, major histocompatibility complex, MVP, MhcVizPipe, NB, nonbinder, QC, quality control, SB, strong binder, WB, weak binder, WSL, Windows Subsystem for Linux

## Abstract

MS-based immunopeptidomics is maturing into an automatized and high-throughput technology, producing small- to large-scale datasets of clinically relevant major histocompatibility complex (MHC) class I-associated and class II-associated peptides. Consequently, the development of quality control (QC) and quality assurance systems capable of detecting sample and/or measurement issues is important for instrument operators and scientists in charge of downstream data interpretation. Here, we created MhcVizPipe (MVP), a semiautomated QC software tool that enables rapid and simultaneous assessment of multiple MHC class I and II immunopeptidomic datasets generated by MS, including datasets generated from large sample cohorts. In essence, MVP provides a rapid and consolidated view of sample quality, composition, and MHC specificity to greatly accelerate the “pass–fail” QC decision-making process toward data interpretation. MVP parallelizes the use of well-established immunopeptidomic algorithms (NetMHCpan, NetMHCIIpan, and GibbsCluster) and rapidly generates organized and easy-to-understand reports in HTML format. The reports are fully portable and can be viewed on any computer with a modern web browser. MVP is intuitive to use and will find utility in any specialized immunopeptidomic laboratory and proteomics core facility that provides immunopeptidomic services to the community.

## Highlights

• MhcVizPipe is a software tool for QC in immunopeptidomics.
• It provides numerical scores for QC analysis of tumor biopsies.
• It performs rapid QC analysis of large clinical immunopeptidomic sample cohorts.
• It generates organized and easy-to-understand reports in HTML format.

## Graphical Abstract

**Figure:**
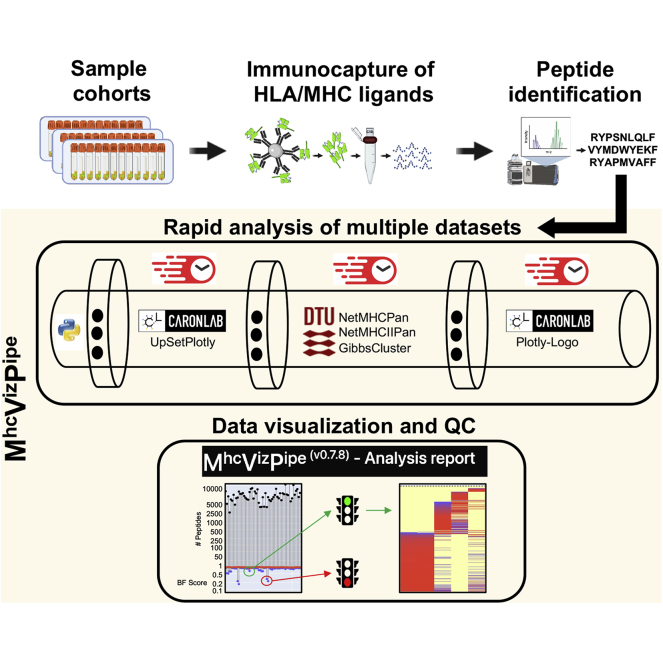

## In Brief

Automated QC software tools capable of detecting sample and/or measurement issues are important for downstream data interpretation. We created MhcVizPipe, the first semiautomated GUI-based QC software tool that enables rapid and simultaneous assessment of multiple MHC class I and II immunopeptidomic datasets generated by MS. MVP is intuitive to use, generate QC reports in HTML formats, and will find utility in any immunopeptidomic laboratory.

The importance of MS-based immunopeptidomics for the discovery of T-cell targets in autoimmunity (1, 2), cancer (3, 4, 5), and infectious diseases (6, 7, 8, 9, 10)—including pandemic pathogens (11, 12)—has attracted the interest of investigators from a wide range of clinical disciplines, leading to the creation of the human immunopeptidome project (13, 14, 15). In fact, the growing interest for clinical immunopeptidomics was recently accelerated by the unquestionable contribution to the 2018 Nobel prize winners James P. Allison and Tasuku Honjo for their work on cancer immunotherapy (16). In addition, the development of next-generation MS technologies and methods has fostered the discovery of new classes of actionable tumor-specific antigens in various cancer types (17, 18, 19, 20, 21, 22). In its simplest form, clinical immunopeptidomics involves the isolation of human leukocyte antigen (HLA) class I-associated and class II-associated peptides from patient biospecimens by immunoaffinity capture, followed by peptide release, and subsequent peptide sequence identification by MS combined with advanced bioinformatics (23). Once the HLA ligands have been confidently identified, their immunogenicity can be evaluated to further guide the development of vaccines and T-cell–based therapies in translational laboratories (24).

Data quality is a cornerstone of solid research, demanding repeatability and reproducibility (25). In that respect, assessing and controlling the quality of immunopeptidomic data generated by MS is of utmost importance. In genomics and MS-based proteomics, the importance of quality control (QC) and quality assurance has been long acknowledged, and various grades of QC samples (*i.e.*, QC1, QC2, and QC3) (26) as well as QC software solutions have been extensively developed and applied over the years (27, 28, 29, 30, 31, 32, 33, 34, 35, 36). In contrast, in MS-based immunopeptidomics, QC samples and QC software tools specialized for major histocompatibility index (MHC) class I-associated and class II-associated peptides remain poorly documented in spite of their importance for successful therapeutic development (37).

To date, only two studies have focused on QC measures to validate the quality of immunopeptidomic data generated by MS (37, 38). In the first study, Ghosh *et al.* (37) described the importance of (i) the mass accuracy of the obtained peptides, (ii) the fitness of the observed and calculated retention times, (iii) the repeatability of the retention times and the signal intensities of the detected peptides, (iv) the use of synthetic peptides (light or heavy) to determine the specificity and limit of detection of the HLA ligands of interest, and (v) the technical and biological reproducibility. The identification scores of the peptides and their identification with different search engines can also be applied to assess data quality (39). In the second QC study, Fritsche *et al.* (38) presented (i) statistics to enable discrimination of true HLA ligands from coisolated HLA-independent proteolytic fragments, (ii) the necessary steps to ensure system suitability of the chromatographic system, (iii) an algorithm for detection of source fragmentation events that are introduced by electrospray ionization during MS, and (iv) an experimental pipeline that enables high-throughput sequence verification through similarity of fragmentation patterns and coelution of synthetic isotope-labeled internal standards. Akin to MS-based proteomics, such QC approaches are useful to show the overall quality of immunopeptidomic datasets in additional to point out limitations and pitfalls that are critical for individual peptides.

Other basic and essential steps to assess the overall quality and MHC specificity of immunopeptidomic datasets are to quantify the total number of peptides per sample, length of the detected peptides, number of strong binders (SBs), weak binders (WBs), and non-MHC binders (NBs) per sample, number of SBs, WBs, and NBs per allele per sample, number of peptides making up each sequence motif, and fraction of each sequence motif attributed to each MHC allele. Current software tools used to assess such QC measures to determine the MHC specificity of immunopeptidomic datasets include MHC peptide-binding prediction algorithms and clustering tools, such as NetMHCpan (40, 41), MHCFlurry (42, 43), GibbCluster (44), and MoDec (45). Although widely used, these algorithms were not purposely built for QC in MS-based immunopeptidomics, and as a result, can process only one sample at a time and generally require further human-based data manipulations (*e.g.*, in Excel)—a relatively time-intensive and error-prone procedure that is not sustainable for QC in large-scale MS-based immunopeptidomics studies, as recently reported (46, 47). Hence, the development of automated or semiautomated QC software tools for rapid and simultaneous quality assessment of the MHC specificity of multiple immunopeptidomic datasets generated by MS has yet to be developed.

**Figure 1:**
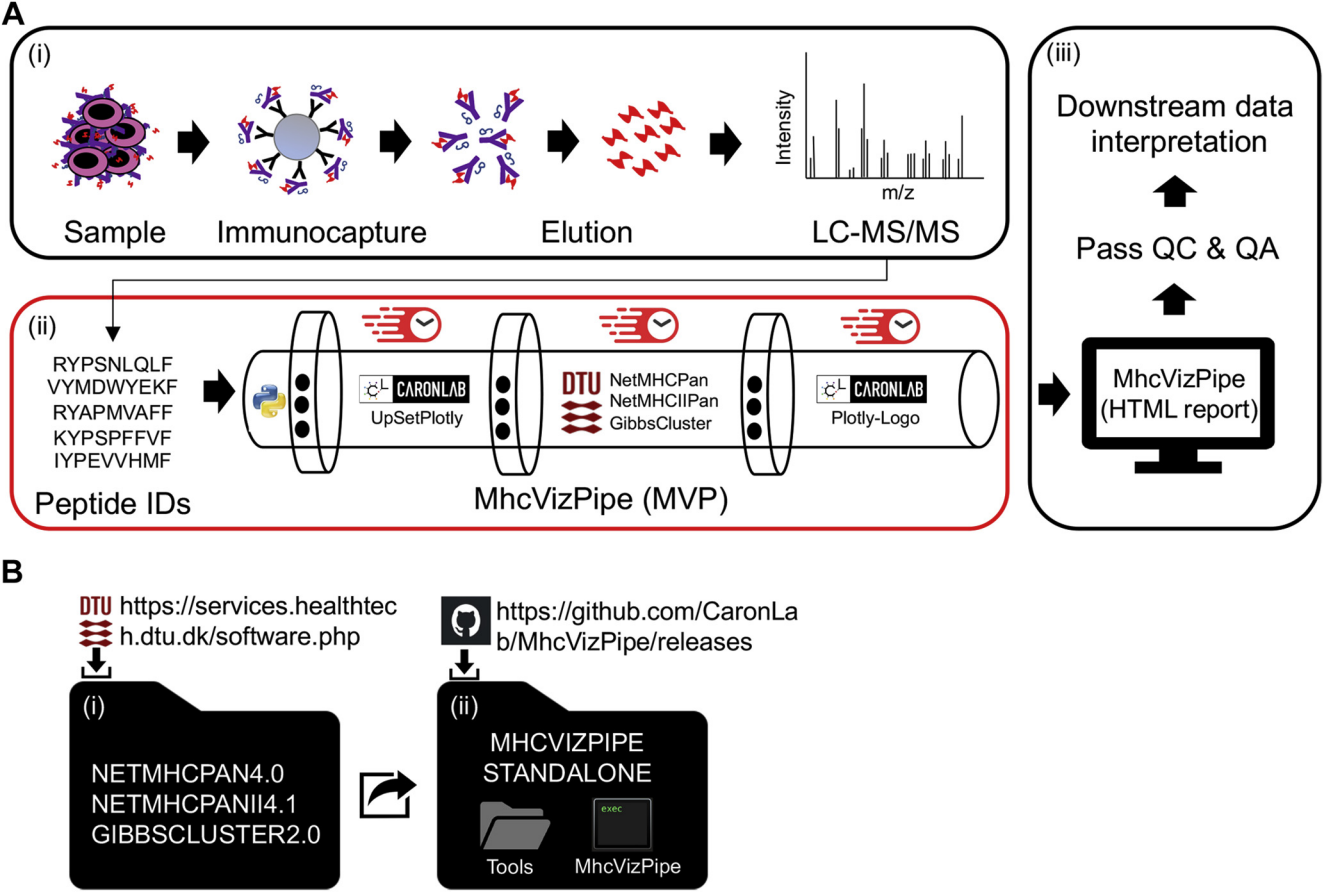
**Overview and installation of the MhcVizPipe (MVP) software.***A*, illustration showing the (i) experimental workflow for the isolation and identification of MHC-associated peptides by LC–MS/MS, (ii) the software tools integrated into the MVP pipeline for rapid data processing, and (iii) the HTML report generated to support (or not) downstream immunopeptidomic data interpretation through quality control (QC) and quality assurance (QA). The MVP pipeline was created with the Python language and parallelizes in-house (UpsetPlotly and Plotly-Logo) and established (NetMHCpan, NetMHCIIpan, and GibbsCluster) algorithms. *B*, installation steps. The user can download and install the MVP software from http://github.com/CaronLab/MhcVizPipe on Linux, Mac, and Windows 10 (using the Windows Subsystem for Linux). The installation requires the download of the third-party software tools and the installation of MVP.

In this technical report, we document QC in MS-based immunopeptidomics by presenting MhcVizPipe (MVP). MVP is an open-source and freely available QC software tool with an intuitive graphical user interface (GUI), intended to be used by any immunopeptidomic laboratory. MVP builds upon the algorithms mentioned previously (NetMHC suite tools and GibbsCluster) and, once installed, provides a semiautomated and fast postdata acquisition system to assess and control sample quality and MHC specificity through effective visualization of one or multiple immunopeptidomic datasets in an HTML report (Fig. 1*A*). Reported QC metrics are peptide length, numbers of MHC binders, distribution of binders per MHC allele, and prominent MHC peptide-binding motifs (see examples of HTML reports in supplemental Data S1–S4). In addition, MVP computes length fraction (LF) scores and binding fraction (BF) scores for each sample to rapidly highlight problematic samples while analyzing large immunopeptidomic sample cohorts. In this regard, a command line interface (CLI) is also available for large-scale/“batch” analyses (https://github.com/CaronLab/MhcVizPipe/wiki/Command-line-interface). Later, we describe (i) the installation procedure of MVP, (ii) the MVP GUI for data upload, (iii) the multiple computational steps that are automatically performed within the MVP-HTML reporting pipeline, (iv) the content of an HTML report, (v) QC analysis of low-quality to high-quality biopsies, (vi) the speed performance of MVP, and (vii) QC analysis of a large cohort composed of 152 samples. We also discuss the current limitations of MVP and how the software could be further developed to support QC in large-scale clinical immunopeptidomic studies.

## Experimental procedures

### System requirements

#### Operating system

MVP runs natively on Linux (*e.g.*, Ubuntu) or MacOS systems. It can also be installed on Windows 10 systems by using the Windows Subsystem for Linux (WSL) to run NetMHCpan, NetMHCIIpan, and GibbsCluster. MVP was tested on the following systems: Ubuntu 16.04, 18.04, and 20.04; Linux Mint 18; MacOS 10.13 HighSierra; 10.14 Mojave; 10.15 Catalina; 11.2.3 Big Sur; and Windows 10 with both WSL 1 and WSL 2.

#### Memory and processor

There are no hard memory or central processing unit requirements for MVP. However, because MVP utilizes multithreading, performance increases on systems with higher numbers of central processing units. The memory usage is minimal and should not present an issue to any recent desktop or laptop computer.

### Components of MVP

MVP connects the bioinformatics tools NetMHCpan, NetMHCIIpan, and GibbsCluster with Python visualization libraries to create portable HTML reports for interpretation of immunopeptidomics MS data. In addition to NetMHCpan, NetMHCIIpan, and GibbsCluster, it makes use of the following third-party Python libraries: Plotly, PlotlyDash, Dash Bootstrap Components, Pandas, Numpy, Dominate, UpsetPlotly, Waitress, and PlotlyLogo (PlotlyLogo and UpsetPlotly developed in-house and described later). The GUI of MVP is built using the PlotlyDash library and runs as a local web application (*i.e.*, it runs in a web browser).

### Peptide list preprocessing

Prior to analysis, peptide lists are stripped of chemical modifications (*e.g.*, oxidation of methionine and carbamidomethylation), any peptides containing nonstandard amino acids are removed, and flanking amino acids are removed if found (*e.g.*, P and V in P.KAPDNRETL.V). It is assumed that flanking amino acids are separated from the main sequence with dots, as indicated in the example. Formats differing from this will require preprocessing by the user.

### Length distributions and intersecting peptide sets

Length distributions are visualized for all peptides ≤30-mer in the input. If multiple samples are analyzed, the intersections of the peptide sequence sets arising from the samples are visualized in an UpSetPlot-type figure (48) using the Python library UpSetPlotly.

### NetMHCpan and NetMHCIIpan

Prior to analysis with NetMHCpan or NetMHCIIpan, subsets of the peptide lists are created as follows: for class I, the lengths are restricted to between 8 and 12 mer inclusive; for class II, the lengths are restricted to between 9 and 22 mer inclusive. These lists are analyzed using either NetMHCpan or NetMHCIIpan to yield binding predictions (eluted ligand [EL] percent rank) used to annotate the peptides as follows: for class I, the rank of SBs is ≤0.5, the rank of WBs is ≤2.0, and the rank of NBs is >2.0; for class II, the rank of SBs is ≤2.0, the rank of WBs is ≤10, and the rank of NBs is >10. The results are presented in tabular format as well as with a bar plot and heatmap. The bar plot shows the number of peptides *versus* aggregate binding strength (*e.g.*, a peptide that has a weak affinity for one allele and a strong affinity for another is counted as an SB). The heatmap is sorted by percent rank EL score from left to right (*i.e.*, the peptides are ordered first by the left-most column, then the second-left-most, and so on). To prevent the convolution of the ordering by poorly scoring peptides, all values greater than 2.5 for class I or 12 for class II are set to 2.5 or 12, respectively. The colormap of the heatmap is set such that red approximately represents SBs, blue approximately represents WBs, and yellow represents NBs with a linear gradient between the colors.

### LF and BF scores

The LF score is the fraction of all peptides that are 8 to 14 mers in length for class I peptides or 8 to 25 mers in length for class II peptides. The BF score is the fraction of all peptides within the above length range, which are predicted to be either WBs or SBs by NetMHCpan or NetMHCIIpan.

### GibbsCluster

MVP performs two GibbsCluster routines, which we have termed “Unsupervised GibbsCluster” and “Allele-Specific GibbsCluster.” There is a tab for each of these in the “Sequence Motifs” section of the report. The unsupervised GibbsCluster is a standard GibbsCluster run using all peptides in the subset described previously. The following parameters are used, which are the recommended defaults for class I and II peptides on the GibbsCluster-2.0 server (https://services.healthtech.dtu.dk/service.php?GibbsCluster-2.0): -g 1-6 -T -j 2 -C -D 4 -I 1 (class I); -g 1-6 -k 1 -T -j 2 (class II). Note that the grouping setting (-g) is actually run one group at a time, as explained in the following section. The grouping with the highest Kullback-Leibler Distance (KLD) score is presented in the report. The “Allele-specific GibbsCluster” is dependent upon the results of NetMHCpan or NetMHCIIpan. A subset of peptides is created for each allele, such that all the peptides in a subset are SBs or WBs for the respective allele. An additional subset is created in which peptides predicted to be NBs for all the alleles are combined (*i.e.*, the peptides in this set are not predicted binders for any allele). Any subset containing less that 20 peptides is discarded. Each of the remaining subsets is run in GibbsCluster using the aforementioned parameters with -g set to 1, forcing GibbsCluster to look for only one peptide group. An exception is the subset of NBs, in which -g is set to 1 to 5, allowing GibbsCluster to look for multiple groups in these unannotated peptides. As aforementioned, the grouping with the highest KLD score is presented in the report. The results are reported in the “Allele-Specific GibbsCluster” tab, where we see one motif for each allele present in the sample, and up to five motifs for the nonbinding peptides. All the peptide groups shown in the “Sequence Motifs” section are visualized using the PlotlyLogo library as described later.

### Multiprocessing in NetMHCpan, NetMHCIIpan, and GibbsCluster

To shorten the overall analysis time, MVP parallelizes the use of NetMHCpan, NetMHCIIpan, and GibbsCluster. Because NetMHCpan and NetMHCIIpan do not take advantage of multiprocessing, in MVP, the peptide lists are broken into smaller lists, which are concurrently analyzed by separate instances of the respective software. The results are then combined back into a single list. GibbsCluster does offer multiprocessing, analyzing the different grouping possibilities (*e.g.*, 1, 2, 3, 4, or 5 groups) concurrently. Unfortunately, with larger peptide lists, the single-group clustering is often long running, which makes it difficult to schedule consecutive GibbsCluster analyses in an efficient manner. To address this, MVP splits the analysis into individual instances of GibbsCluster, each analyzing only a single grouping possibility. For example, instead of using the command line parameter “-g 1-5” as indicated in the previous sections, in actuality, MVP creates five jobs with the parameters -g 1, -g 2, -g 3, and so on. With a single sample, this does not result in a speed improvement, but with multiple samples, MVP is able to efficiently schedule the jobs from multiple samples to utilize all available processors.

### UpSetPlotly

UpSetPlotly is an open-source Python package, based upon UpSet (48), developed in-house for visualizing intersecting sets using the Plotly Python library. The source code is available at https://github.com/kevinkovalchik/UpSetPlotly, and it can be installed as as “upsetplotly” from PyPI using the Pip package manager.

### PlotlyLogo

PlotlyLogo is an open-source Python package developed in-house for generating sequence logos from sequence alignment data and is available at https://github.com/kevinkovalchik/Plotly-Logo. PlotlyLogo was designed for the specific purpose of generating sequence logos from sequence alignments as native Python objects using the Plotly plotting framework. As such, the version used in this report (plotly-logo, version 0.0.2) does not include much of the functionality of more complete solutions such as Seq2Logo, but it has the advantage of utilizing a modern Python framework (compatible with Python3) and of generating figures as Python objects, which can be directly used in other Python code. The choice to develop PlotlyLogo rather than using an existing Python solution such as LogoMaker (49) was influenced by the desire to generate live and interactive figures in a portable HTML format. It is developed in the Python programming language and requires the Python plotting library Plotly. It is installable as “plotly-logo” from PyPI using the Pip package manager. The algorithms in PlotlyLogo are based upon the methods described by Thomsen *et al.* for Seq2Logo (50) and in Immunological Bioinformatics (51). In brief, sequence alignments are read from text-formatted files, and probability matrices are generated using sequence weighting from Hobohm 1 clustering and pseudocount correction, as described (50, 51). Two types of sequence logos can be generated: Shannon and Kullback–Leibler.

### Datasets

To test the performance of EL *versus* binding affinity (BA) in annotating peptide for specific MHC alleles, the following datasets were used: H2-K^b^ and H2-D^b^ class I peptides extracted from mouse liver tissue (PXD008733) (52), HLA-A and HLA-B class I peptides extracted from peripheral blood mononuclear cell (PXD001872) (39), H2-IA^d^ and H2-IE^d^ class II peptides extracted from the mouse A20 cell line (53), and HLA-DQA10101, -DQB10501 and HLA0DQA10103, -DQB10603 class II peptides extracted from the human MAVER-1 cell line (54). To test the speed performance of MVP, the following datasets were used: HLA-ABC-associated peptides extracted from the JY cell line and multiple peripheral blood mononuclear cell samples (PXD001872) (39) and H2-K^b^/H2-D^b^ class I peptides extracted from various mouse tissues (PXD008733) (52). Supplemental data in the study by Rijensky *et al.* (55) and Shraibman *et al.* (46) were used to calculate the QC scores.

### Time speed estimation procedure

To benchmark the speed performance of MVP, the selected datasets were processed manually (human based) or using MVP (computer based). Time estimates were measured using a stopwatch. For the computer-based approach, the data were processed with MVP using an iMac18,2 (MacOS Big Sur, version 11.2.3) with four cores and 16 GB memory. For the human-based approach, the exact same datasets were processed manually by an experienced researcher with expertise in immunopeptidomics, with the goal of generating figures and tables that are produced in an HTML report. In other words, the human-based approach mimicked as much as possible the computer-based approach to produce the equivalent of an HTML report. Hence, for the human-based approach, time estimates were measured using a stopwatch for the following actions: (1) make graphs and tables for peptide length distribution and specificity in Microsoft Excel, (2) run NetMHCpan 4.1 and GibbsCluster online for each dataset, (3) extract output files and manipulate the data manually for making histograms and heatmaps in Microsoft Excel from the predicted MHC peptide BA scores, and (4) combine the results in a document.

### Batch analysis

To facilitate automation or batch analysis—which might benefit from running MVP in a bash script, a python script, or as a scheduled process—MVP contains a CLI. The CLI runs MVP and saves the HTML report, NetMHCpan predictions, and PDF files of all figures in the report in a specified location. The use of the CLI is described at the following link: https://github.com/CaronLab/MhcVizPipe/wiki/Command-line-interface.

## Results

### Installation of MVP

MVP is freely available at https://github.com/CaronLab/MhcVizPipe and can be downloaded and installed on Linux (*e.g.*, Ubuntu), Mac, and Windows 10 (using the Windows Subsystem for Linux). A detailed explanation of the installation process can be found on the MVP GitHub wiki: https://github.com/CaronLab/MhcVizPipe/wiki. Note that for installing and running MVP on Windows 10, a few additional steps are required (see https://github.com/CaronLab/MhcVizPipe/wiki/Windows-installation for details). In brief, two installation options are available: (1) a ZIP file containing a standalone Python distribution, and all the Python packages required by MVP can be downloaded from the MVP GitHub repository (https://github.com/CaronLab/MhcVizPipe/releases) and (2) MVP can be installed from the Python Package Index into an existing Python environment. Both options also require the separate acquisition of copies of NetMHCpan-4.1, NetMHCIIpan-4.0, and GibbsCluster-2.0 from DTU Health Tech (https://services.healthtech.dtu.dk/software.php). Note that when new versions of NetMHCpan and GibbsCluster will be made publicly available, users will need to download the new versions themselves as there is no possibility at this time to automatically update the tools. When new versions are available, we will ensure that MVP functions with them.

Option 1 consists of a ZIP file that contains MVP, a standalone Python distribution, and all the required Python packages. The user must then copy or move the extracted DTU Health Tech tools (NetMHCpan, NetMHCIIpan, and GibbsCluster) into the “tools” folder found inside the MVP directory (Fig. 1*B*). MVP can then be run by double clicking the MVP executable file or running the accompanying MhcVizPipe.sh script file. This installation option does not require any use of the terminal, and the final program is fully portable (*e.g.*, you could put it on a USB drive and use it on any compatible computer, though use in Windows requires you to copy MVP from the USB to the hard drive before use).

While we have tried to ensure the portable installation is compatible with most systems, we cannot guarantee it will work on every computer. This stems from the fact that the standalone distribution of Python has system dependencies that are usually, but not always, present. To address this issue, it is also possible to install MVP into an existing Python environment as described in “option 2.”

Option 2 is intended for users who are familiar with the command line and Python and wish to install the MVP Python package themselves or who are unable to use the portable distribution for the reasons mentioned previously. MVP can be installed in an existing Python environment (≥3.7) from the Python Package Index by invoking “pip install MhcVizPipe” from the terminal. As aforementioned, the user must also acquire copies of NetMHCpan-(4.0 or 4.1), NetMHCIIpan-4.0, and GibbsCluster-2.0. For option 2, the user needs to ensure that the tools from DTU Health Tech are properly configured (according to their accompanying documentation) and reference their locations in the MVP settings window after starting the program for the first time. With this type of installation, the program is started from a terminal using this command: “python -m MhcVizPipe.gui.”

Because MVP is run as a web server, it can be accessed by any computer connected to the local network, facilitating use by multiple users. If unexpected issues are encountered during the installation procedure, please contact the authors or open an issue at the following link: https://github.com/CaronLab/MhcVizPipe/issues. The proper installation of MVP can be tested using example peptide lists and accompanying HTML reports available at the following link: https://github.com/CaronLab/MhcVizPipe/tree/master/test_data.

### The MVP GUI

**Figure 2:**
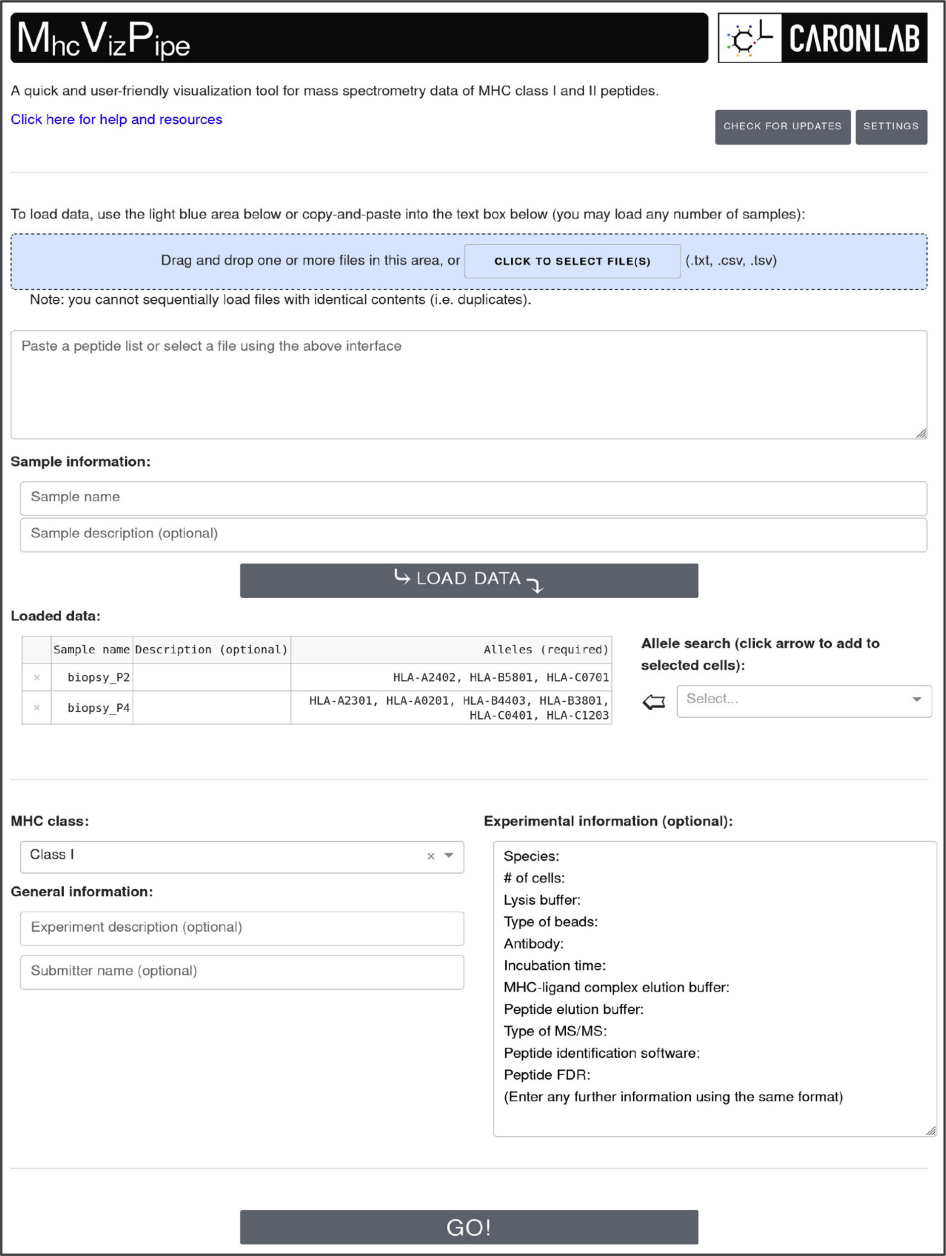
**Overview of the MhcVizPipe (MVP) graphical user interface (GUI).** To run an analysis, the user can copy–paste a list of peptides or upload more than one file at a time. Samples can be labeled with detailed information. The user click the “GO!” button to start an analysis, and a loading screen appears while the analysis is running, followed by a pop-up window with a link to the HTML report. More details about the GUI are available at https://github.com/CaronLab/MhcVizPipe/wiki/Usage. A command line interface (CLI) is also available for “batch” analyses at https://github.com/CaronLab/MhcVizPipe/wiki/Command-line-interface.

MVP provides a simple and intuitive GUI in any web browser (tested in Firefox, Safari, Chrome, Chromium, and Edge) (Fig. 2). Peptide lists can be uploaded in different formats (.csv, .tsv, or .txt) or copy-pasted into the GUI. Any file of the mentioned formats may be loaded provided they have columns headers or are a simple list. If a multicolumn file is opened (*e.g.*, database search results in .csv format), the user is asked by MVP to select the header of the column that contains the peptide sequences. There is no limit to the number of files that can be analyzed at one time, though processing time increases with the number of files and the interpretability of figures and tables will decrease with excessive numbers of samples. Once the peptide lists have been uploaded, the MHC class (I or II) and alleles corresponding to the sample(s) are specified. Up to six alleles may be specified for each sample. Many technical details related to the samples can also be specified and will appear in the final report, which is generated in a portable HTML format and can be viewed in the browser or saved to the computer. MVP also generates zip files containing editable PDF versions of all plots in the report and all the data used to generate the report (*i.e.*, NetMHCpan predictions, GibbsCluster output files).

### Automated steps performed within the MVP reporting pipeline

Peptide lists are stripped of peptides containing chemical modifications. The complete lists are then used for generating the length histogram and UpSet plot in the “Sample overview” section of the HTML report. The lists are then subset to peptides 8 to 12 or 9 to 22 mers in length for class I or class II peptides, respectively. Binding predictions for these subsets are made using NetMHCpan4.0 or 4.1 or NetMHCIIpan4.0 for each user-indicated allele, and the EL percent rank scores are used to generate the “Annotation Results” and “Binding Heatmaps” sections in the HTML report. LF and BF scores are also calculated to provide numerical values regarding the overall quality of the samples. LF score is the fraction of all peptides that are 8 to 12 mers in length for class I peptides or 9 to 22 mers in length for class II peptides; BF score is the fraction of all peptides within the appropriate length range, which are predicted to be either WBs or SBs by NetMHCpan or NetMHCIIpan (Table 1). Peptide grouping and alignment is then performed by GibbsCluster twice, once using the complete subset of peptides (yielding the results in the “Unsupervised GibbsCluster” tab) and then again for the sets of peptides predicted to bind the different alleles (yielding the results in the “Allele-Specific GibbsCluster” tab). Logos of the most prominent motifs identified by GibbsCluster (according to KLD scores) are generated using Plotly-Logo. Note that the goal of this clustering approach is not to provide new biological insights or highly accurate annotations of peptide subgroups to their alleles but to rather provide an overall assessment of the quality and MHC specificity of the data.

**Table 1:** LF and BF scores calculated from immunopeptidomic data generated from eight different tumor biopsies

Sample	Cancer type	Sample weight (mg)	Total peptides	Peptides (8–12 mers)	LF score	BF score
Biopsy 1	Head and neck (adnexal adenocarcinoma)	<13	352	284	0.81	0.62
Biopsy 2	Bile duct (cholangiocarcinoma)	60	144	68	0.47	0.07
Biopsy 3	Lung (N/A)	120	486	207	0.43	0.44
Biopsy 4	Gastric (carcinoma)	100	969	452	0.47	0.32
Biopsy 5	Vascular (hemangioendothelioma)	<13	691	585	0.85	0.93
Biopsy 6	Head and neck (squamous cell carcinoma)	<13	1018	969	0.95	0.98
Biopsy 7	Bladder (sarcomatoid carcinoma)	<13	391	323	0.83	0.92
Biopsy 8	Pancreatic (adenocarcinoma)	150	981	897	0.7	0.89

Abbreviation: N/A, not available.

Immunopeptidomic data were obtained from the study by Rijensky *et al*. Eight different biopsies and cancer types were analyzed. The sample weight, total number of peptides identified, fraction of peptides between 8 and 12 mers, and LF and BF scores are indicated. LF and BF scores are color coded to illustrate high-quality (*green*), middle-quality (*brown–red*), and low-quality (*red*) immunopeptidomic data.

### MVP annotates peptides using the EL method

NetMHCpan (version 4.1) and NetMHCIIpan (version 4.0) are critical components of the MVP software for scoring class I and II peptides, respectively (Fig. 1*A*). The predicted MHC BA computed by these algorithms defines SBs (%Rank <0.5), WBs (2.0 < %Rank >0.5), or NBs (%Rank >2.0) in the immunopeptidome dataset. Those predicted values are used to calculate the BF scores, which serve as an index of MHC specificity for the samples.

The methods that are available for peptide scoring in NetMHCpan and NetMHCIIpan are EL and BA (41). Here, we compared both methods using four publicly available immunopeptidomic datasets (see Experimental procedures section) to rationalize which method to apply and integrate as part of the MVP software. For class I peptides, we found that EL and BA performed equally well (supplemental Fig. S1). Indeed, the two methods resulted in very similar numbers of SBs and WBs, both in mouse (supplemental Fig. S1, *A* and *B*) and human (supplemental Fig. S1, *C* and *D*). In contrast, for class II peptides, substantial differences were observed between EL and BA, in both mouse (supplemental Fig. S2, *A* and *B*) and human (supplemental Fig. S2, *C* and *D*). Specifically, we found that EL resulted in a nearly 4-fold increase in annotated peptides compared with BA. Furthermore, we found that the peptide groups identified by GibbsCluster contained a much higher proportion of SBs when using EL (up to 75%) *versus* BA (up to 14%) (supplemental Fig. S2, *B* and *D*). These results are in agreement with a recent study indicating that prediction scores based on EL were more accurate than those using BA, for class II peptides in particular (56). Thus, the EL method was chosen and implemented in MVP.

### Content of the HTML report

The HTML report generated from the MVP GUI contains three main sections: (1) sample overview, (2) annotation results and binding heatmaps, and (3) sequence motifs. Examples of HTML reports are provided in supplemental Data S1 and S2 for mouse and human class I peptides, respectively; and in supplemental Data S3 and S4 for mouse and human class II peptides, respectively.

The first section of the HTML report (Sample overview) contains (i) a peptide length distribution graph (up to 30-mers), (ii) a descriptive table indicating the total number of peptides per sample, the number of peptides corresponding to the expected length related to the MHC class selected, and LF and BF scores for each sample, and (iv) an “UpSet” plot (48) showing the number of unique and shared peptides between multiple samples.

In the second section of the HTML report (*annotation results and binding heatmaps*), MVP uses the EL scoring and annotation method described previously to generate histograms and heatmaps illustrating the proportion of immunoaffinity-purified peptides predicted to bind MHC molecules. Those graphs provide a bird's eye view on the quality and MHC specificity of the samples and are a way to visualize the BS scores calculated in the “Sample overview” section.

The third section of the HTML report focuses on the identification and visualization of sequence motifs that are enriched in the analyzed samples using GibbsCluster and our in-house Plotly-Logo algorithm, respectively (see the Experimental procedures section). The sequence motifs from the GibbsCluster analysis are presented in two ways: (i) the unsupervised tab displays the most prominent motif(s) represented by the subset of peptides in the sample along with the percent of peptides associated with each allele and (ii) the allele-specific cluster tab displays peptide motif(s) generated from SBs and WBs for each allele, as well as motifs from peptides predicted to be nonassigned MHC binders. Together, MVP generates in a few clicks a complete HTML report for assessing the quality and MHC specificity of immunopeptidomic data generated by MS.

### QC analysis of tumor biopsies

**Figure 3:**
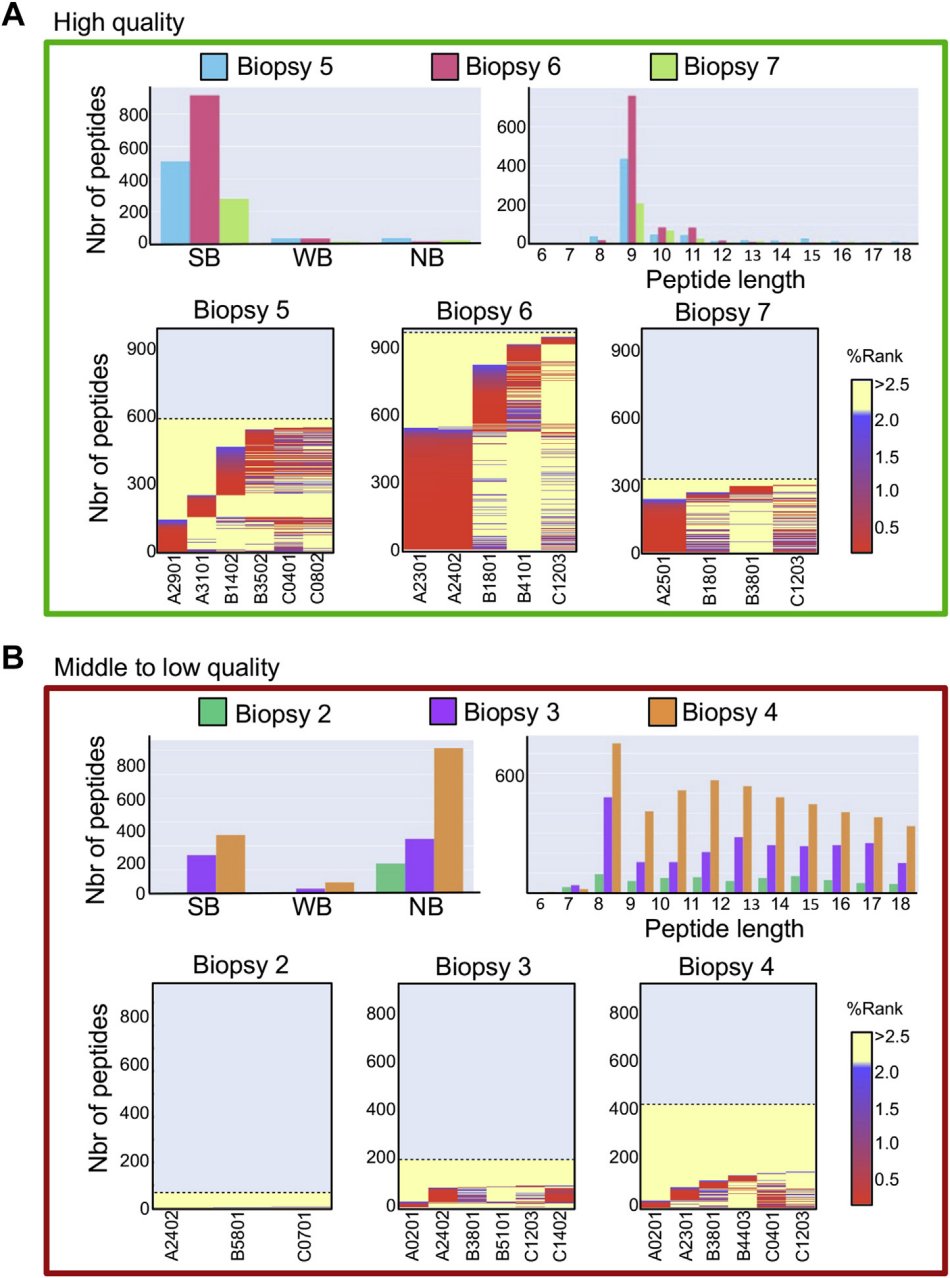
**Visualization of the QC scores (LF and BF) is calculated in**Table 1**.***A* and *B*, histograms and heatmaps illustrating high-quality (*A*) and middle- to low-quality (*B*) immunopeptidomic data generated by MS from various tumor biopsies (Table 1). Histograms showing the distribution of peptides according to their length (*right panel*) and predicted MHC binding affinity (*left panel*). Heatmaps are automatically generated by MVP and provided in the HTML report. For each HLA allele, NetMHCpan provides a %Rank score for individual peptides, which are color coded on the heatmap in *red*, *blue*, and *yellow* for SB, WB, and NB, respectively, with a linear gradient between the colors. BF, binding fraction; HLA, human leukocyte antigen; LF, length fraction; MHC, major histocompatibility complex; MVP, MhcVizPipe; NB, nonbinder; QC, quality control; SB, strong binder; WB, weak binder.

The general quality and MHC specificity of immunopepitdomic data are assessed using LF and BF scores, as mentioned previously. Such numerical values are useful to rapidly detect abnormalities to determine if samples are of high, middle, or low quality. To show the utility of this scoring approach, we selected a small immunopeptidomic dataset generated from eight representative tumor biopsies, recently published in the study by Rijensky *et al.* (55). This dataset is composed of biopsies of different cancer types, including head and neck, bile duct, lung, gastric, vascular, bladder, and pancreatic cancer. Sample weight of those biopsies ranged from <13 to 150 mg, and number of peptides detected ranged from 144 peptides (bile duct; cholandiocarninoma) to 1018 peptides (head and neck; squamous cell carcinoma) (Table 1). To calculate the LF scores, the number of peptides between 8 and 12 mers per biopsy sample was divided by the total number of peptides detected. To calculate the BF score, the number of peptides predicted to be SB or WB (NetMHCpan %Rank) were divided by the number of peptides between 8 and 12 mers. Our data show that the LF scores varied from 0.43 to 0.95 and the BF scores varied from 0.06 to 0.98 (Table 1)—a numerical value of 1 being the highest score. Overall, those samples were qualified of low to high quality. For instance, biopsies 5, 6, and 7 yielded high-quality immunopeptidomic data, with an average combined score (LF and BF) of 0.91. Indeed, the proportions of peptides between 8 and 12 mers that were predicted to bind the HLAs expressed in these biopsy samples were relatively high, as visualized in Figure 3*A*. In contrast, biopsy 2 yielded low-quality immunopeptidomic data, with an average combined score (LF and BF) of 0.27 (Table 1). This relatively low score can be explained by the mere absence of 8 to 12 mers predicted as SB or WB (Fig. 3*C*). Finally, biopsies 3 and 4 yielded middle-quality immunopeptidomic data, with an average combined score (LF and BF) of 0.42 (Table 1). This score can be explained by the noticeable presence of SB and WB within a relatively large proportion of peptides that were observed to be longer than 12 mers (likely contaminant peptides), and therefore, not predicted to bind the HLAs expressed in these biopsy samples (Fig. 3*B*). Thus, our analysis show that LF and BF is a simple QC scoring approach that is integrated within the MVP software tool to determine the overall quality and MHC specificity of HLA peptidomes isolated from human biopsies of various cancer types.

### High-speed performance of MVP

**Figure 4:**
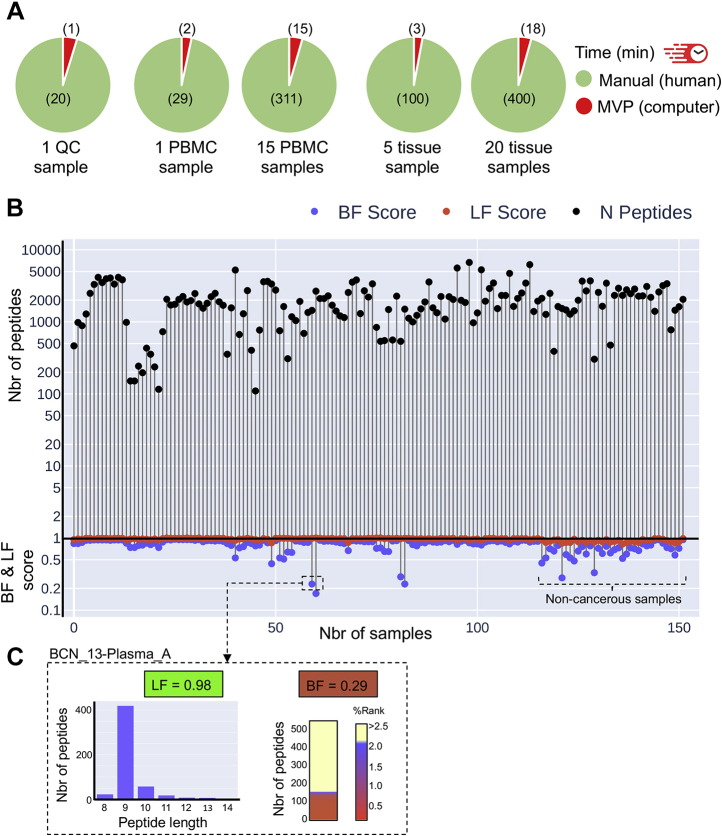
**Speed performance of the MVP software for small- to large-scale QC analyses.***A*, Pie charts showing the time estimates to process and analyze the selected datasets manually (human-based in *green*) or using MVP (computer-based in *red*). The following datasets were tested (from *left to right*): one QC sample (JY), one PBMC sample, 15 PBMC samples, five mouse tissue samples, and 20 mouse tissue samples. *B*, large-scale QC analysis of the HLA peptidome of 152 samples. Graph showing the total number of peptides and the two scores calculated based on peptide lengths (LF) and the number of peptides predicted to bind HLAs (BF) (*y*-axis). Each sample has three points, one for each of the values (shown in the legend). The points for each sample are connected by a *vertical line*. *C*, zoom in on the sample “BCN_13-Plasma_A.” Histogram and heat map showing the length distribution and predicted HLA-binding affinity (%Rank) of the detected peptides in this sample. LF and BF scores are indicated. BF, binding fraction; HLA, human leukocyte antigen; LF, length fraction; MVP, MhcVizPipe; PBMC, peripheral blood mononuclear cell; QC, quality control.

A major advantage of processing immunopeptidomic data through MVP is the speed at which HTML reports are generated for one or multiple samples. To benchmark the speed performance of MVP, we measured the precise time estimates to assess data quality of five different datasets using (i) the human/manual-based approach, as currently done in many laboratories or (ii) the computer/MVP-based approach (see the Experimental procedures section) (Fig. 4*A*). Briefly, the commonly applied human-based approach includes uploading peptide lists and running multiple web interfaces, downloading the results, editing them in Excel, making multiple figures, and then copying them into a reporting document. The computer/MVP-based approach includes uploading peptide lists on the GUI, selecting the appropriate alleles, and clicking the “GO!” button (Fig. 2). The datasets that were selected to benchmark the software included a range of different samples per dataset (Fig. 4*A*). By comparing the two approaches, our results show that the measured time estimates varied from ∼20 min (for one sample) to ∼400 min (for 20 mouse tissue samples) using the conventional human/manual-based approach. Notably, the time estimates for the exact same datasets varied from 1 min (for one sample) to 18 min (for 20 mouse tissue samples) using the computer-/MVP-based approach, thereby accelerating the analysis and figure/table generation process by ∼22-fold on average (Fig. 4*A*). Thus, MVP represents a QC software package in MS-based immunopeptidomics and provides unprecedented speed for fast visualization and quality assessment of immunopeptidomic data. Given its high-speed performance, MVP should find utility in QC analysis of large immunopeptidome sample cohorts.

### QC analysis of a large immunopeptidomic sample cohort

To assess the performance of MVP to run “batch” analysis, we selected a large immunopeptidomic dataset recently published in the study by Shraibman *et al.* (46). In this study, Shraibman *et al.* (46) reported the analysis of the HLA immunopeptidome of 152 samples, including (i) plasma-soluble HLA molecules of 142 plasma samples from both glioblastoma and noncancerous patients and (ii) membranal HLA of 10 glioblastoma tumor tissues. This analysis covers 52 different HLA allotypes, more than 35,000 different HLA-associated peptides, and represents, to the best of our knowledge, the largest immunopeptidomic dataset ever generated from plasma samples. The “batch analysis” of the 152 samples was run automatically using the CLI and a simple Python script (https://github.com/CaronLab/MhcVizPipe/wiki/Command-line-interface). The run time was approximately 81 min (running in Ubuntu 20.04 on an 8-core desktop). The metrics files generated when running MVP from the command line (sample_metrics.txt) were used to generate a QC plot showing three points/scores per sample (*i.e.*, the total number of peptides detected and the LF and BF scores) and illustrating the variability in these scores between all the samples (Fig. 4*B*). In this dataset, the average LF score was 0.96 and the average BF score was 0.80, highlighting the overall high quality of this large dataset. In addition, the generated QC plot was particularly useful to point out potentially problematic samples. For instance, among the lowest BF scores in this dataset was from the sample BCN_13-Plasma_A (Fig. 4*C*). In fact, visualization of the data from this sample indicates that 151 peptides were predicted to be SB or WB, whereas 376 peptides were predicted to be NB, hence, resulting in a relatively low BF score of 0.29 (Fig. 4*C*). Thus, MVP can run “batch” analysis relatively rapidly to provide an assessment of the quality of individual samples within a large immunopeptidomic dataset. Together, MVP is a generic software tool, can be applied to any HLA immunopeptidomes, and given its high-speed performance and scalability, will find utility to rapidly warn the user about problematic samples in large-scale clinical immunopeptidomic studies.

## Discussion

The development of QC software is common practice for high-quality research in the life sciences. Here, we present MVP, a GUI-based QC approach, to rapidly and simultaneously assess the quality and MHC specificity of small to large immunopeptidomic datasets generated by MS. Although MVP can be perceived as a relatively basic software solution by experts in the field, this is, to the best of our knowledge, one of the first reports focusing on the development of QC software in MS-based immunopeptidomics. Indeed, very little has been described in the literature with regard to the development of QC approaches, despite the fact that QC will become critically important in MS-based immunopeptidomics to comply with pharmaceutical regulations for real-life clinical applications (37).

For various reasons, the development of QC software tools has not been prioritized in immunopeptidomics over the last recent years. The main reason is most likely because most immunopeptidomic studies still have to deal with relatively small-scale datasets; and the quality and MHC specificity of such datasets can still be manually assessed using the currently available peptide sequence clustering and MHC peptide-binding algorithms. However, with the recent development of automated technologies for robust and high-throughput MHC immunoprecipitation protocols, the production of immunopeptidomic data on the scale of multiple gigabytes per instrument per day can be anticipated to accelerate the pace of capturing therapeutically relevant data (57). In this regard, at least two large-scale immunopeptidomics studies have recently been reported: (1) the profiling of immunopeptidomes from 152 clinical biospecimens, which were used in this study (46) and (2) the mapping of the first draft of the human immunopeptidome from 227 benign tissue samples, for a total of nearly 3500 raw files generated by MS (47). With this perspective in mind, one can envision that other large-scale immunopeptidomic cohort studies will be conducted in the future, as recently discussed in the context of the human immunopeptidome project (14). Therefore, we believe that automated QC software solutions specialized in MS-based immunopeptidomics are inevitable. The creation of MVP represents a very first step in this direction and should stimulate the development of additional QC software solutions and products specialized in MS-based immunopeptidomics in the future.

The current version of MVP has obvious limitations. MVP was not designed to provide new biological insights. MVP is intended so far for situations where instrument operators or scientists in charge of downstream data analysis want a quick and consolidated view of the general quality, composition, and MHC specificity of one or multiple immunopeptidomic samples in parallel. Whether the user should continue using the evaluated data or not (*e.g.*, with low-quality data) will very much depend on the goal of the project or the specific scientific question(s) asked. Therefore, we think that it is up to the user to judge what is best, if he and/or she should drop low-quality samples or keep troubleshooting them to perform additional experiments and analyses.

MVP is also subject to the accuracy of the available MHC peptide prediction algorithms and clustering tools. In this regard, new algorithms continue to be released for the prediction of HLA class I peptides (42, 43, 58) and HLA class II peptides (45, 59) and will be integrated in future versions of MVP in a yearly basis to improve the overall performance of the QC software. The predictors used in the future to upgrade MVP will also improve its accessibility. In the current version of MVP, NetMHCpan suite tools were used, although they remain poorly compatible with Windows. Therefore, installing MVP on Windows is possible, as explained in detailed in GitHub (https://github.com/CaronLab/MhcVizPipe/wiki/Windows-installation) but is more challenging for a noncomputer expert. Integration of Windows-compatible HLA peptide-binding algorithms, such as MHCFlurry, will help to improve the accessibility of future versions of MVP.

Over the last recent years, considerable advances in HLA ligand purification protocols, MS instrumentations, and computational methods have pushed forward the boundaries of MS-based immunopeptidomics. For instance, state-of-the-art data-independent acquisition methods have been applied (39, 60, 61), specialized computational workflows have been created (62, 63), and new automated and semiautomated HLA ligand purification platforms have been developed to increase the throughput, speed, sensitivity, and reproducibility of immunopeptidome analysis by MS (57, 64, 65). In the context of this progressive technological development, the current version of MVP will inevitably need to be further developed to reach its full QC capabilities and potential for the field. Currently, MVP is best suited for peptide datasets generated by data-dependent acquisition-MS. However, a long-term goal in immunopeptidomics is to scale to large and quantitative clinical datasets generated by data-independent acquisition-MS (39, 60, 61), and subject to stringent QC, as is performed in large-scale clinical proteomics studies (66, 67, 68, 69, 70). In this regard, generation of high-quality and quantitative immunopeptidomic data in large-scale studies is challenging because of issues induced by the samples themselves because of collection, storage, and processing. To help overcome this challenge, MVP could be further developed to evaluate immunopeptidomic data in near real-time—as described in proteomics (27)—allowing for interventions as soon as deviations in data quality of specific immunopeptidomic samples are detected.

Beside QC software, we also believe that future community-driven immunopeptidomic studies that focus on the evaluation and establishment of QC samples/standards are needed. In fact, QC samples in immunopeptidomics remain poorly documented to date (37). Nevertheless, such samples are critical in the long run to generate reproducible clinical datasets across multiple laboratories (13). If successful in doing so, robust and high-quality immunopeptidomic data would be consistently generated, stored, and shared through specialized public repositories—for example, SysteMHC Atlas (71, 72) and caAtlas (73)—thereby providing a global and sustainable mechanism to foster collaborations among computational scientists, biostatisticians, immunologists, and clinical investigators to improve T-cell–based immunotherapies (13).

In conclusion, MVP is a semiautomated GUI-based QC software for rapid assessment of multiple immunopeptidomic datasets generated by MS. The current version of MVP parallelizes the use of well-established immunopeptidomic algorithms as well as new in-house software tools to assess data quality and MHC specificity at unprecedented speed and will therefore be of immediate utility for any expert and nonexpert in the field. We envision that further development of this software tool will facilitate QC for upcoming large-scale immunopeptidomic cohort studies.

## Data availability

All the data generated or analyzed during this study are included in this published article and/or the supplemental data. Created datasets and code are publicly available. Immunopeptidomic data visualized in supplemental Data S2 have been deposited to the ProteomeXchange Consortium (http://proteomecentral.proteomexchange.org) *via* the PRIDE partner repository with the dataset identifier PXD028633. All the codes are available at github: https://github.com/CaronLab/MhcVizPipe.

The MVP source code is open source and freely available at: https://github.com/CaronLab/MhcVizPipe. The version of MVP used in this publication is 0.7.9 (https://github.com/CaronLab/MhcVizPipe/releases/tag/v0.7.9).

## Supplemental data

This article contains supplemental data (39, 52, 53).

## Conflict of interest

A. J. and E. P. are employees of CellCarta (Montreal, Canada); M. T., L. R., and R. B. are employees of Biognosys (Zürich, Switzerland). All other authors declare no competing interests.
