# Supplementary material for: MhcVizPipe: A Quality Control Software for Rapid Assessment of Small- to Large-Scale Immunopeptidome Datasets
Source: Mol Cell Proteomics. 2021 Nov 17;21(1):100178. doi: 10.1016/j.mcpro.2021.100178 (PMC8717601; doi:10.1016/j.mcpro.2021.100178)
Supplement: Supplemental data S1 [file mmc1.zip › mcpro_100178_mmc1.html]

MhcVizPipe Report


# M

### hc

# V

### iz

# P

### ipe

##### (v0.7.8)

### - Analysis report

---

**Date:** 2021-09-30

**Submitted by:** Anonymous

**Analysis type:** Class I

**Description of experiment:**

Sci Data Caron et al.

**Samples:**

SPLEEN:
Alleles: H-2-Kb
LIVER:
Alleles: H-2-Kb
INTESTINE:
Alleles: H-2-Kb
LUNG:
Alleles: H-2-Kb
THYMUS:
Alleles: H-2-Kb

**Species:** MOUSE

**Type of beads:** CNBR

---

### Sample Overview

- LF Score: fraction of peptides between 8 and 12 mers.
- BF Score: fraction of peptides between 8 and 12 mers which are predicted to be strong or weak binders.

| Sample | Total peptides | Peptides between 8-12 mers | LF Score | BF Score |
| --- | --- | --- | --- | --- |
| SPLEEN | 2430 | 2287 | 0.94 | 0.79 |
| LIVER | 3860 | 3429 | 0.89 | 0.65 |
| INTESTINE | 7532 | 5763 | 0.77 | 0.29 |
| LUNG | 2005 | 1819 | 0.91 | 0.66 |
| THYMUS | 1694 | 1574 | 0.93 | 0.73 |

**UpSet Plot** (only displaying intersections containing >= 1% of at least one sample)

**Peptide Length Distribution** (maximum of 30 mers)

---

### Annotation Results

NetMHCpan eluted ligand predictions made for all peptides between 8 & 12 mers, inclusive.
- Percent rank cutoffs for strong and weak binders: 0.5 and 2.0.
- Percentages are calculated across rows (i.e. percentage of total peptides for a respective sample).

| Sample | Total peptides | Allele | Strong binders | Weak binders | Non-binders |
| --- | --- | --- | --- | --- | --- |
| SPLEEN | 2287 | H-2-Kb | 1660 (72.6%) | 157 (6.9%) | 470 (20.6%) |
| LIVER | 3429 | H-2-Kb | 1923 (56.1%) | 300 (8.7%) | 1206 (35.2%) |
| INTESTINE | 5763 | H-2-Kb | 1393 (24.2%) | 250 (4.3%) | 4120 (71.5%) |
| LUNG | 1819 | H-2-Kb | 1092 (60.0%) | 112 (6.2%) | 615 (33.8%) |
| THYMUS | 1574 | H-2-Kb | 1078 (68.5%) | 78 (5.0%) | 418 (26.6%) |

**Binding Affinities**

---

### Binding Heatmaps

NetMHCpan eluted ligand predictions made for all peptides between 8 & 12 mers, inclusive.
Approximate color legend (detailed mapping shown next to heatmaps):

Predicted strong binders (%rank <= 0.5)

Predicted weak binders (0.5 < %rank <= 2.0)

Predicted non-binders

**- -** # of peptides in sample

---

### Sequence Motifs

Clustering performed with all peptides between 8 & 12 mers, inclusive.

- Percentages represent the percentage of peptides in a given group predicted to strongly bind the indicated allele.

Polar

Neutral

Basic

Acidic

Hydrophobic

- Unsupervised GibbsCluster
- Allele-specific GibbsCluster

**SPLEEN** (peptides clustered: 2287, outliers: 333)

Peptides in group: 1954

**H-2-Kb: 85%**

**LIVER** (peptides clustered: 3429, outliers: 795)

Peptides in group: 2634

**H-2-Kb: 73%**

**INTESTINE** (peptides clustered: 5763, outliers: 286)

Peptides in group: 641

**H-2-Kb: 1%**

Peptides in group: 1531

**H-2-Kb: 79%**

Peptides in group: 670

**H-2-Kb: 14%**

Peptides in group: 810

**H-2-Kb: 9%**

Peptides in group: 900

H-2-Kb: 0%

Peptides in group: 925

H-2-Kb: 0%

**LUNG** (peptides clustered: 1819, outliers: 403)

Peptides in group: 1416

**H-2-Kb: 77%**

**THYMUS** (peptides clustered: 1574, outliers: 313)

Peptides in group: 1261

**H-2-Kb: 85%**

**SPLEEN sequence motif(s)**

**H-2-Kb**

Peptides: 1788

**Non-binders group 2**

Peptides: 207

**Non-binders group 1**

Peptides: 162

**LIVER sequence motif(s)**

**H-2-Kb**

Peptides: 2172

**Non-binders group 2**

Peptides: 513

**Non-binders group 1**

Peptides: 426

**INTESTINE sequence motif(s)**

**H-2-Kb**

Peptides: 1609

**Non-binders group 2**

Peptides: 1783

**Non-binders group 1**

Peptides: 1627

**LUNG sequence motif(s)**

**H-2-Kb**

Peptides: 1188

**Non-binders group 2**

Peptides: 248

**Non-binders group 1**

Peptides: 228

**THYMUS sequence motif(s)**

**H-2-Kb**

Peptides: 1142

**Non-binders group 2**

Peptides: 155

**Non-binders group 1**

Peptides: 169
