# Supplementary material for: MhcVizPipe: A Quality Control Software for Rapid Assessment of Small- to Large-Scale Immunopeptidome Datasets
Source: Mol Cell Proteomics. 2021 Nov 17;21(1):100178. doi: 10.1016/j.mcpro.2021.100178 (PMC8717601; doi:10.1016/j.mcpro.2021.100178)
Supplement: Supplemental data S2 [file mmc2.zip › mcpro_100178_mmc2.html]

MhcVizPipe Report


# M

### hc

# V

### iz

# P

### ipe

##### (v0.7.8)

### - Analysis report

---

**Date:** 2021-09-30

**Submitted by:** Anonymous

**Analysis type:** Class I

**Description of experiment:**

Data from ProteomeXchange PXD028633

**Samples:**

JY:
Alleles: HLA-A0201, HLA-B0702, HLA-C0702

**Species:** human

**Antibody:** w6/32

---

### Sample Overview

- LF Score: fraction of peptides between 8 and 12 mers.
- BF Score: fraction of peptides between 8 and 12 mers which are predicted to be strong or weak binders.

| Sample | Total peptides | Peptides between 8-12 mers | LF Score | BF Score |
| --- | --- | --- | --- | --- |
| JY | 6986 | 6705 | 0.96 | 0.95 |

**Peptide Length Distribution** (maximum of 30 mers)

---

### Annotation Results

NetMHCpan eluted ligand predictions made for all peptides between 8 & 12 mers, inclusive.
- Percent rank cutoffs for strong and weak binders: 0.5 and 2.0.
- Percentages are calculated across rows (i.e. percentage of total peptides for a respective sample).

| Sample | Total peptides | Allele | Strong binders | Weak binders | Non-binders |
| --- | --- | --- | --- | --- | --- |
| JY | 6705 | HLA-A0201 | 1918 (28.6%) | 252 (3.8%) | 4535 (67.6%) |
|  |  | HLA-B0702 | 3839 (57.3%) | 563 (8.4%) | 2303 (34.3%) |
|  |  | HLA-C0702 | 456 (6.8%) | 1554 (23.2%) | 4695 (70.0%) |

**Binding Affinities**

---

### Binding Heatmaps

NetMHCpan eluted ligand predictions made for all peptides between 8 & 12 mers, inclusive.
Approximate color legend (detailed mapping shown next to heatmaps):

Predicted strong binders (%rank <= 0.5)

Predicted weak binders (0.5 < %rank <= 2.0)

Predicted non-binders

**- -** # of peptides in sample

---

### Sequence Motifs

Clustering performed with all peptides between 8 & 12 mers, inclusive.

- Percentages represent the percentage of peptides in a given group predicted to strongly bind the indicated allele.

Polar

Neutral

Basic

Acidic

Hydrophobic

- Unsupervised GibbsCluster
- Allele-specific GibbsCluster

**JY** (peptides clustered: 6705, outliers: 196)

Peptides in group: 4271

HLA-A0201: 0%,

**HLA-B0702: 89%,** 

HLA-C0702: 3%

Peptides in group: 2238

**HLA-A0201: 85%,** 

HLA-B0702: 2%,

HLA-C0702: 12%

**JY sequence motif(s)**

**HLA-A0201**

Peptides: 2157

**HLA-B0702**

Peptides: 4370

**HLA-C0702**

Peptides: 1955

**Non-binders group 2**

Peptides: 147

**Non-binders group 1**

Peptides: 119
