# Supplementary material for: MhcVizPipe: A Quality Control Software for Rapid Assessment of Small- to Large-Scale Immunopeptidome Datasets
Source: Mol Cell Proteomics. 2021 Nov 17;21(1):100178. doi: 10.1016/j.mcpro.2021.100178 (PMC8717601; doi:10.1016/j.mcpro.2021.100178)
Supplement: Supplemental data S3 [file mmc3.zip › mcpro_100178_mmc3.html]

MhcVizPipe Report


# M

### hc

# V

### iz

# P

### ipe

##### (v0.7.8)

### - Analysis report

---

**Date:** 2021-09-30

**Submitted by:** Anonymous

**Analysis type:** Class II

**Description of experiment:**

DATA FROM https://doi.org/10.1002/pmic.201700246

**Samples:**

MAVER-1\_DQ:
Alleles: HLA-DQA10103-DQB10603, HLA-DQA10101-DQB10501

**Species:** HUMAN

---

### Sample Overview

- LF Score: fraction of peptides between 9 and 22 mers.
- BF Score: fraction of peptides between 9 and 22 mers which are predicted to be strong or weak binders.

| Sample | Total peptides | Peptides between 9-22 mers | LF Score | BF Score |
| --- | --- | --- | --- | --- |
| MAVER-1\_DQ | 2452 | 2354 | 0.96 | 0.83 |

**Peptide Length Distribution** (maximum of 30 mers)

---

### Annotation Results

NetMHCIIpan eluted ligand predictions made for all peptides between 9 & 22 mers, inclusive.
- Percent rank cutoffs for strong and weak binders: 2.0 and 10.0.
- Percentages are calculated across rows (i.e. percentage of total peptides for a respective sample).

| Sample | Total peptides | Allele | Strong binders | Weak binders | Non-binders |
| --- | --- | --- | --- | --- | --- |
| MAVER-1\_DQ | 2354 | HLA-DQA10103-DQB10603 | 1282 (54.5%) | 538 (22.9%) | 534 (22.7%) |
|  |  | HLA-DQA10101-DQB10501 | 584 (24.8%) | 529 (22.5%) | 1241 (52.7%) |

**Binding Affinities**

---

### Binding Heatmaps

NetMHCIIpan eluted ligand predictions made for all peptides between 9 & 22 mers, inclusive.
Approximate color legend (detailed mapping shown next to heatmaps):

Predicted strong binders (%rank <= 2.0)

Predicted weak binders (2.0 < %rank <= 10.0)

Predicted non-binders

**- -** # of peptides in sample

---

### Sequence Motifs

Clustering performed with all peptides between 9 & 22 mers, inclusive.

- Percentages represent the percentage of peptides in a given group predicted to strongly bind the indicated allele.

Polar

Neutral

Basic

Acidic

Hydrophobic

- Unsupervised GibbsCluster
- Allele-specific GibbsCluster

**MAVER-1\_DQ** (peptides clustered: 2354, outliers: 168)

Peptides in group: 2186

**HLA-DQA10103-DQB10603: 58%,** 

HLA-DQA10101-DQB10501: 26%

**MAVER-1\_DQ sequence motif(s)**

**HLA-DQA10103-DQB10603**

Peptides: 1799

**HLA-DQA10101-DQB10501**

Peptides: 1103

**Non-binders group 2**

Peptides: 163

**Non-binders group 1**

Peptides: 192
