# Supplementary material for: MhcVizPipe: A Quality Control Software for Rapid Assessment of Small- to Large-Scale Immunopeptidome Datasets
Source: Mol Cell Proteomics. 2021 Nov 17;21(1):100178. doi: 10.1016/j.mcpro.2021.100178 (PMC8717601; doi:10.1016/j.mcpro.2021.100178)
Supplement: Supplemental data S4 [file mmc4.zip › mcpro_100178_mmc4.html]

MhcVizPipe Report


# M

### hc

# V

### iz

# P

### ipe

##### (v0.7.8)

### - Analysis report

---

**Date:** 2021-09-30

**Submitted by:** Anonymous

**Analysis type:** Class II

**Description of experiment:**

Data from Sofron et al. https://doi.org/10.1002/eji.201545930

**Samples:**

A20\_:
Alleles: H-2-IAd, H-2-IEd

**Species:** Mouse

---

### Sample Overview

- LF Score: fraction of peptides between 9 and 22 mers.
- BF Score: fraction of peptides between 9 and 22 mers which are predicted to be strong or weak binders.

| Sample | Total peptides | Peptides between 9-22 mers | LF Score | BF Score |
| --- | --- | --- | --- | --- |
| A20\_ | 1329 | 1319 | 0.99 | 0.88 |

**Peptide Length Distribution** (maximum of 30 mers)

---

### Annotation Results

NetMHCIIpan eluted ligand predictions made for all peptides between 9 & 22 mers, inclusive.
- Percent rank cutoffs for strong and weak binders: 2.0 and 10.0.
- Percentages are calculated across rows (i.e. percentage of total peptides for a respective sample).

| Sample | Total peptides | Allele | Strong binders | Weak binders | Non-binders |
| --- | --- | --- | --- | --- | --- |
| A20\_ | 1319 | H-2-IAd | 926 (70.2%) | 207 (15.7%) | 186 (14.1%) |
|  |  | H-2-IEd | 65 (4.9%) | 231 (17.5%) | 1023 (77.6%) |

**Binding Affinities**

---

### Binding Heatmaps

NetMHCIIpan eluted ligand predictions made for all peptides between 9 & 22 mers, inclusive.
Approximate color legend (detailed mapping shown next to heatmaps):

Predicted strong binders (%rank <= 2.0)

Predicted weak binders (2.0 < %rank <= 10.0)

Predicted non-binders

**- -** # of peptides in sample

---

### Sequence Motifs

Clustering performed with all peptides between 9 & 22 mers, inclusive.

- Percentages represent the percentage of peptides in a given group predicted to strongly bind the indicated allele.

Polar

Neutral

Basic

Acidic

Hydrophobic

- Unsupervised GibbsCluster
- Allele-specific GibbsCluster

**A20\_** (peptides clustered: 1319, outliers: 86)

Peptides in group: 1233

**H-2-IAd: 75%,** 

H-2-IEd: 4%

**A20\_ sequence motif(s)**

**H-2-IAd**

Peptides: 1125

**H-2-IEd**

Peptides: 286

**Non-binders group 1**

Peptides: 137
