# Supplementary material for: MhcVizPipe: A Quality Control Software for Rapid Assessment of Small- to Large-Scale Immunopeptidome Datasets
Source: Mol Cell Proteomics. 2021 Nov 17;21(1):100178. doi: 10.1016/j.mcpro.2021.100178 (PMC8717601; doi:10.1016/j.mcpro.2021.100178)
Supplement: Supplemental Figures S1 and S2 [file mmc5.pdf]

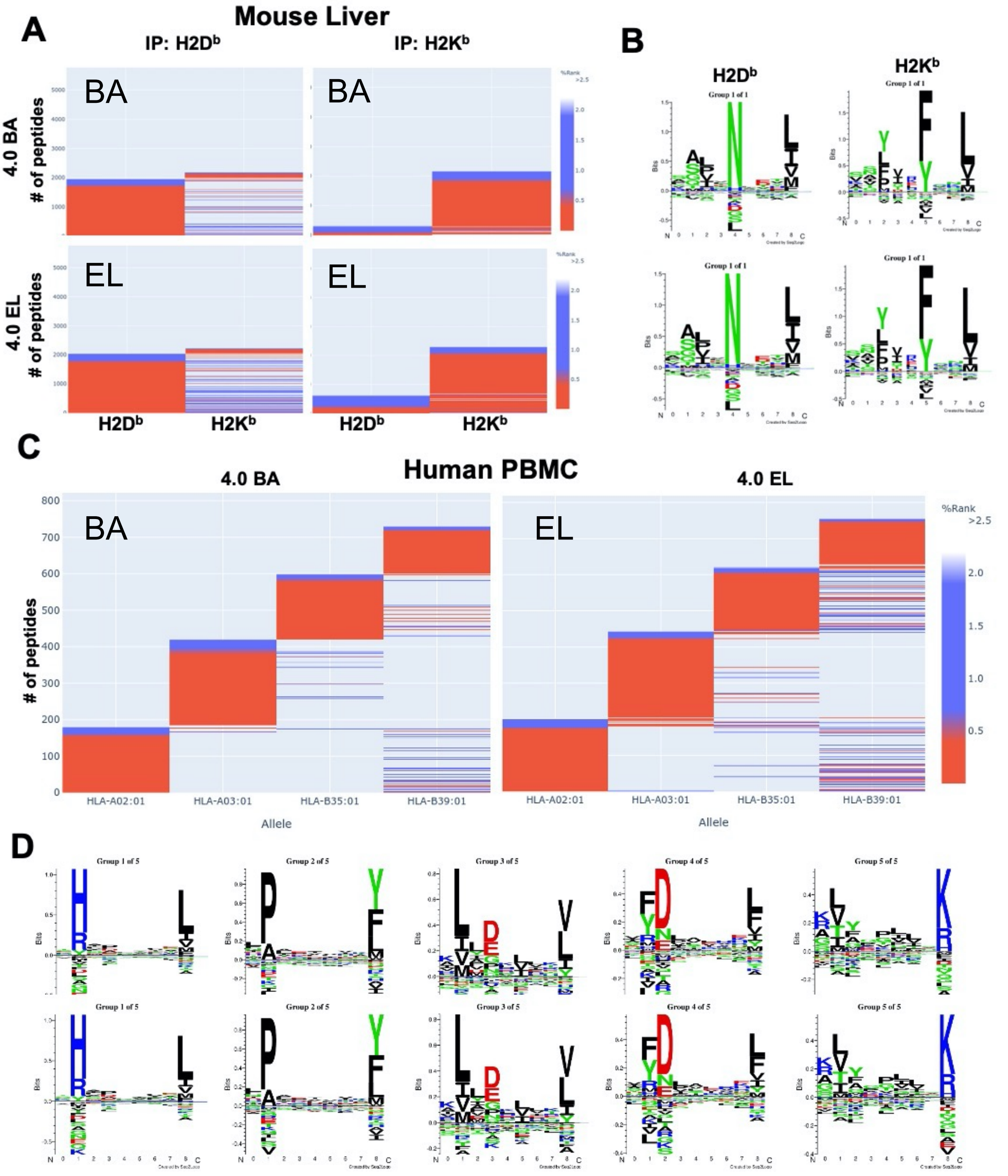

Supplemental Figure 1. Comparison of EL and BA scoring from NetMHCpan4.0 for class I peptides. Heatmaps and sequence motifs of mouse liver (**A, B**) and human PBMC (**C, D**) samples show absence of differences between the two scoring systems for class I peptides. Mouse data were used from https://doi.org/10.1038/sdata.2018.157 (52) and human data were used from https://doi.org/10.7554/eLife.07661.001 (39).

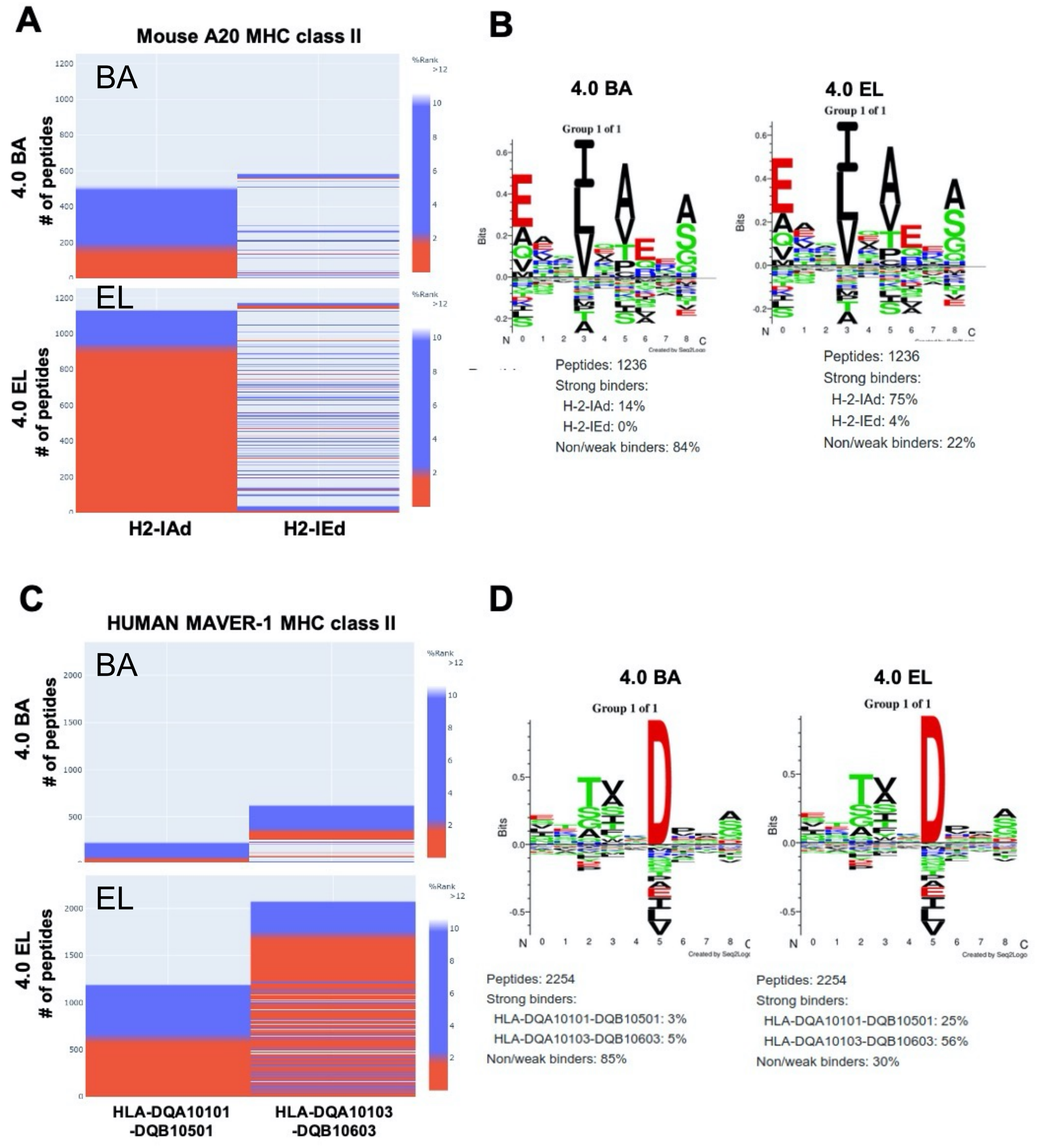

Supplemental Figure 2. Comparison of EL and BA scoring from NetMHCIIpan4.0 for class II peptides. Heatmaps and sequence motifs of the mouse A20 cell line (**A, B**) and the human MAVER-1 cell line (**C, D**) samples show significant differences between the two scoring systems for class II peptides. The EL score increases the number of MHC class II binders by approximately 4-fold over BA scoring. Published mouse data were used from https://doi.org/10.1002/eji.201545930 (52) and human data were used from https://doi.org/10.1002/pmic.201700246 (53).
